# Chemical composition of heartwood and sapwood of *Tectona grandis* characterized by CG/MS-PY

**DOI:** 10.1038/s41598-022-22800-1

**Published:** 2022-11-02

**Authors:** Vinícius Resende de Castro, Paula Gabriella Surdi, Sergio Antonio Fernandes, Matheus da Silva Berger, Antonio José Vinha Zanuncio, José Cola Zanuncio, Solange de Oliveira Araujo

**Affiliations:** 1 Departamento de Engenharia Florestal, Universidade Federal de Viçosa, Viçosa, MG 36570-900 Brazil; 2 Instituto de Ciências Agrárias, Universidade Federal de Uberlândia, Monte Carmelo, MG 38500-000 Brazil; 3 Centro de Estudos Florestais, Instituto Superior de Agronomia, Universidade de Lisboa, 1349-017 Lisboa, Portugal

**Keywords:** Natural variation in plants, Plant physiology

## Abstract

Teak wood has chemical compounds that can be used for pharmaceutical and textile industries, in addition, this compounds are related to resistance to biodeterioration, color and modification processes. Heartwood and sapwood of *T. grandis* (teak), 15 years-old, were characterized by Py-CG/MS analysis and syringyl (S)/guaiacyl (G) ratio was evaluated. Heartwood and sapwood were pyrolyzed at 550 °C and 62 and 51 compounds were identified from them, respectively. The acetic acid (10%) and levoglucosan (26.65%) were the most abundant compound in the sapwood and heartwood, respectively. The high acetic acid content enhances the use of teak wood to production of artificial essences for perfumery, paints, dyes. While levoglucosan can be used in the manufacture of epoxy resins, antiparasitic and insecticides. The organic compounds identified include 2-methylanthraquinone as one of the main component responsible for the resistance of the teak wood to biological factors (fungi and termites). The syringyl (S)/guaiacyl (G) ratio of heartwood and sapwood was 0.51 and 0.50, respectively.

## Introduction

The high dimensional stability and natural durability of the *Tectona grandis* (Verbenaceae) wood characterizes this plant as one of the most important in the world^1,2^. Native to Southeast Asia (Burma, India, Myanmar, Thailand etc.), *T. grandis* are introduced and adapted to Central (Belize, Panama and Puerto Rico) and South America (Brazil, Colombia, Guyanas and Venezuela)^3^.

Brazil has more than 90,000 hm^2^ of planted teak forests, 40% growth in ten years, this wood is mainly used in the naval industry and furniture manufacturing. However, the national market is very restricted, studies on the chemical composition can enable the use of teak wood for other purposes^4,5^. These compounds are mainly quinones like the anthraquinones group conferring resistance to xylophagous organisms, but with variations in composition and concentration related to age, genetic material, tree position (heartwood and sapwood), silvicultural treatments, soil, climate and management^6^.

Teak extractives can be used as bioproducts, antioxidants and compounds with antitumor activity and teak lignin in adhesives, paints, polymers and resinsand^3,7^. The large amount of the quinone group, such as 2-methyl-9,10-anthraquinone (tectoquinone), 1,5-dihydroxy-2-methyl-9,10-anthraquinone and lapachol, 2-hydroxy-3-(3 -methyl-2-butenyl)-1, and 4-naphthoquinone potentiate teak wood for natural dyes production^7^. The composition of lignins is alto important, high values of the synringyl/guaiacyl ratio result in lignin with higher reactivity and low resistance to thermal decomposition^8^.

Analytical pyrolysis is a technique to characterize materials in the absence of oxygen by chemical degradation reactions induced by thermal energy^9^. This technique uses a set of small molecular species, separated by gas chromatography and segregating products from pyrolysis^10^.

Data obtained from analysis by gas chromatography, coupled with mass spectrometry and analytical pyrolysis (Py-GC/MS), allow to identify and to classify compounds in different samples^11^.

The objective of the study was to identify, by Py-GC/MS, compounds obtained and the syringyl/guaiacyl ratio of heartwood and sapwood from 15-year-old *Tectona grandis* and use chemical characterization to indicate potential uses for teak wood.

## Methods

Three 15-year-old *T. grandis* trees were harvested in commercial plantations with a spacing of 3 × 3 m in the municipality of Nova Maringá, Mato Grosso state, Brazil (coordinates of 13° 00′ 55.08″ S and 57° 05′ 52.48″ O). This experiment included the collection of plant material, complied with the national laws of Brazil, respecting the Brazilian environmental code. Four discs at the DBH height (1.30 m from the ground) were removed per tree and the heartwood and sapwood regions marked by visual analysis and crushed separately. The 40–60 mesh fraction from these materials was subjected to pyrolysis in Py-GC/MS using the Pyrolyzer Single-Shot “PY-3030S (Frontier Lab Inc, Fukushima, Japan) connected to a GC-2010 Plus system with a GCMS-QP2010 Ultra (Shimadzu, Kyoto, Japan). The temperature of the valve interface was set at 290 °C and the pyrolysis temperature at 500 °C. The GC column used was an Rtx-5MS (Restek), 30 m long, 0.25 mm internal diameter and 0.25 µm thick. The temperature of GC injector was adjusted to 250 °C, the initial oven temperature at 40 °C for 1 min and the heating rate was 6 °C/min until reaching 280 °C with the samples remaining for 15 min at this temperature. Helium was the carrier gas at 1 mL/min flow rate in split mode and then at a split ratio of 20:1. The mass of the samples subjected to pyrolysis was 0.5 mg. The peaks of the compounds, obtained after the pyrolysis of the samples, were identified based on the NIST and Wiley mass spectral data library. The yield of the products formed was obtained dividing the area of each peak by the total area (sum of all areas of the compounds in the chromatogram).

The syringyl/guaiacyl ratio was calculated using the area values of the signals recorded in the pyrogram and compared with the result obtained by the method of alkaline oxidation with nitrobenzene^12^. The S/G ratio was obtained by the ratio between the concentration of syringaldehyde and that of vanillin considering the group of markers guaiacol; 4-methyl-1,2-benzenediol; 4-vinyl guaiacol; vanillin; eugenol; isoeugenol; as the guaiacyl type and the methoxyeugenol compounds; cis-4-propenylsyringol; syringaldehyde; 4-vinylsyringol and trans-4-propenylsyringol as the syringyl type.

## Results and discussion

The pyrolytic analysis detected 85 compounds in the wood, being 51 in the sapwood and 61 in the heartwood (Table 1). The acetic acid (10%) was the most abundant compound in the sapwood followed by the oxiranemethanol (5.88%), phenol, 4-ethenyl-2-methoxy (7.71%), propanoic acid, 2-oxo (5.51%), levoglucosan (4.47%), 1,2-cyclopentanedione (4.08%) and isoeugenol (4%), with the other compounds representing less than 3.22% of the total. In the heartwood, the most abundant compounds were levoglucosan (26.65%), acetic acid (6.34%), cyclohexanecarboxaldehyde, 4-methoxy (5.1%), squalene (4.85%) and isoeugenol (3. 65%) with less than 2.97% for the others. Two and four compounds from sapwood and heartwood, respectively, were not identified. Acetic acid, the main component of sapwood, can be used in the manufacture of inks, dyes, synthetic silks, cellulose acetate and medicines^13^, while levoglucosan, the main component with heartwood, can be used in the manufacture of epoxy resins, antiparasitic and insecticides^14^. Many compounds found are phenolic, these components are fundamental in the resistance against attack by xylophagous agents and can be used in the chemical and food industry^3^.

**Table 1:** Area of the pyrolytic compounds peaks (percentage of the total chromatogram area) of the teak sapwood and heartwood.

Peak	Compound	Sapwood	Heartwood	
1	Acetic acid, oxo	5.51	–	
2	1,2-Propanediamine	–	2.79	
3	Oxiranemethanol	5.88	2.97	
4	Butanoic acid, 3-hydroxy	1.87	–	
5	Diethylene oximide	3.08	–	
6	Propanal, 2,3-dihydroxy	–	0.68	
7	Acetic acid	10.70	6.34	
8	Propanoic acid, 2-oxo	5.51	0.91	
9	D-Glucitol, 1,4:3,6-dianhydro	0.29	–	
10	D-Limonene	0.22	–	
11	NI	–	0.59	
12	2-Propanone, 1-hydroxy	3.07	2.38	
13	Butanedial	1.55	–	
14	2-Pentanone, 4-methyl	2.15	–	
15	Methyl pyruvate	–	2.12	
16	Furfural	–	0.97	
17	NI	0.95	–	
18	2-Furancarboxaldehyde	–	0.11	
19	2-Butanone	0.15	–	
20	2-Furanmethanol	0.24	–	
21	Octanal	0.42	–	
22	Octaldehyde	–	0.81	
23	1,2-Cyclopentanedione	4.08	–	
24	Cyclopentanone, 2-methyl	–	0.97	
25	NI	0.26	1.53	
26	Cyclohexanecarboxaldehyde, 4-methoxy	–	5.10	
27	NI	0.71	–	
28	1,2-Cyclopentanedione, 3-methyl-	0.81	–	
29	NI	0.33	–	
30	Nonanaldehyde	1.09	1.89	
31	Guaiacol	2.96	–	
32	2-Isopropylcyclohexanol	–	1.07	
33	Pentanal	2.52	–	
34	Dodecanoic acid, 3-hydroxy	0.11	0.35	
35	Phenol, 2-methoxy-4-methyl	1.87	1.67	
36	2-Pentanol, 5-(2-propynyloxy)	–	1.76	
37	3,4-Dimethoxytoluene	0.20	–	
38	Trans-2-Decenal	1.21	2.17	
39	p-Ethylguaiacol	0.45	–	
40	Cyclohexanol, 4-isopropyl	–	0.35	
41	4-Ethylcyclohexanol	0.18	–	
42	2,4-Decadienal	0.61	1.01	
43	Phenol, 4-ethenyl-2-methoxy	4.71	1.45	
44	2-Undecenal	1.31	1.78	
45	Eugenol	0.61	0.17	
46	Syringol	2.38	0.72	
47	Dodecanoic acid, 3-hydroxy	0.11	0.21	
48	Phenol, 2-methoxy-5-(1-propenyl)	0.39	–	
49	Vanillin	0.77	0.62	
50	Isoeugenol	4.00	3.65	
51	Tetradecane	0.16	–	
52	Toluene, 3,4,5-trimethoxy	0.43	–	
53	Guaiacylacetone	0.68	–	
54	3',5'-Dimethoxyacetophenone	3.22	2.08	
55	Tridecanaldehyde	2.73	0.46	
56	Levoglucosan	4.47	26.65	
57	Phenol, 2,6-dimethoxy-4-(2-propenyl)	0.27	–	
58	Syringaldehyde	0.78	1.05	
59	Methoxyeugenol	2.60	2.63	
60	Acetosyringone	0.56	1.20	
61	4-Hydroxy-2-methoxycinnamaldehyde	1.03		
62	Desaspidinol	0.53	0.37	
63	Ethanone, 1-[2-(5-hydroxy-1,1-dimethylhexyl) -3-methyl-2-cyclopropen-1-yl]	–	0.55	
64	2(3H)-Benzofuranone, hexahydro-4,4,7a-trimethyl-	–	0.19	
65	Pentadecanoic acid	–	0.36	
66	Oleylaldehyde	0.16	0.23	
67	Octadecanal	0.15	–	
68	Oleic Acid	0.26	–	
69	Myristaldehyde	–	0.94	
70	Palmitic acid	1.20	1.43	
71	Cyclopentadecanone, 2-hydroxy	–	2.56	
72	Heneicosane	0.14	–	
73	NI	0.86	–	
74	3,5-Dimethoxy-4-hydroxycinnamaldehyde	–	0.12	
75	Hexacosane	0.10	–	
76	Vinyl myristate	–	0.22	
77	Anthraquinone, 2-methyl	–	1.63	
78	Octadecanoic acid	3.07	0.47	
79	2-Hydroxycyclopentadecanone	–	2.88	
80	Stearic acid	0.30	–	
81	Octadecanal	0.15	–	
82	Stearaldehyde	0.14	–	
83	Benzylidenepinacolone	–	0.55	
84	1,1'-Biphenyl-3,4,4'-trimethoxy-6'-formyl	0.97	0.56	
85	Squalene	1.87	4.85	

*NI* The compound was not identified.

The number of compounds identified in the sapwood and heartwood in the 15-year-old teak wood was lower than that reported for this plant with 70-year-old plant, with 127 compounds identified, being 74 and 71% in sapwood and heartwood, respectively^15^. In *Quercus* spp. wood, 40 different compounds were found, the acetic acid, levoglucosan, 3/2-furaldehyde and 2,3-dihydro-5-methylfuran-2-one were the main products^16^. This may be due to age (15- and 70-year-old) and pyrolysis temperature differences between these works. Compounds with the largest area are mainly derived from carbohydrates and found in the initial part of the pyrogram with shorter retention time, and the lignin and extractives derivates in the middle and at the end of the pyrogram with a smaller area^17^. The ion-intensive mass spectra of lignin derivatives correspond, in most cases, to the base peak and the compounds with carbohydrates are more easily fragmented into smaller ones (except levoglucosan) giving, in general, low molecular mass ions and difficult to identify. Some compounds identified in the chromatogram are similar to those obtained from teak wood between 10^6^ and 70^15^ years old subjected to a thermochemical process^18,19^. This similarity of some components is due to the higher concentration of components (lignin derivatives), which are more stable when subjected to the thermochemical process^19^.

**Figure 1:**
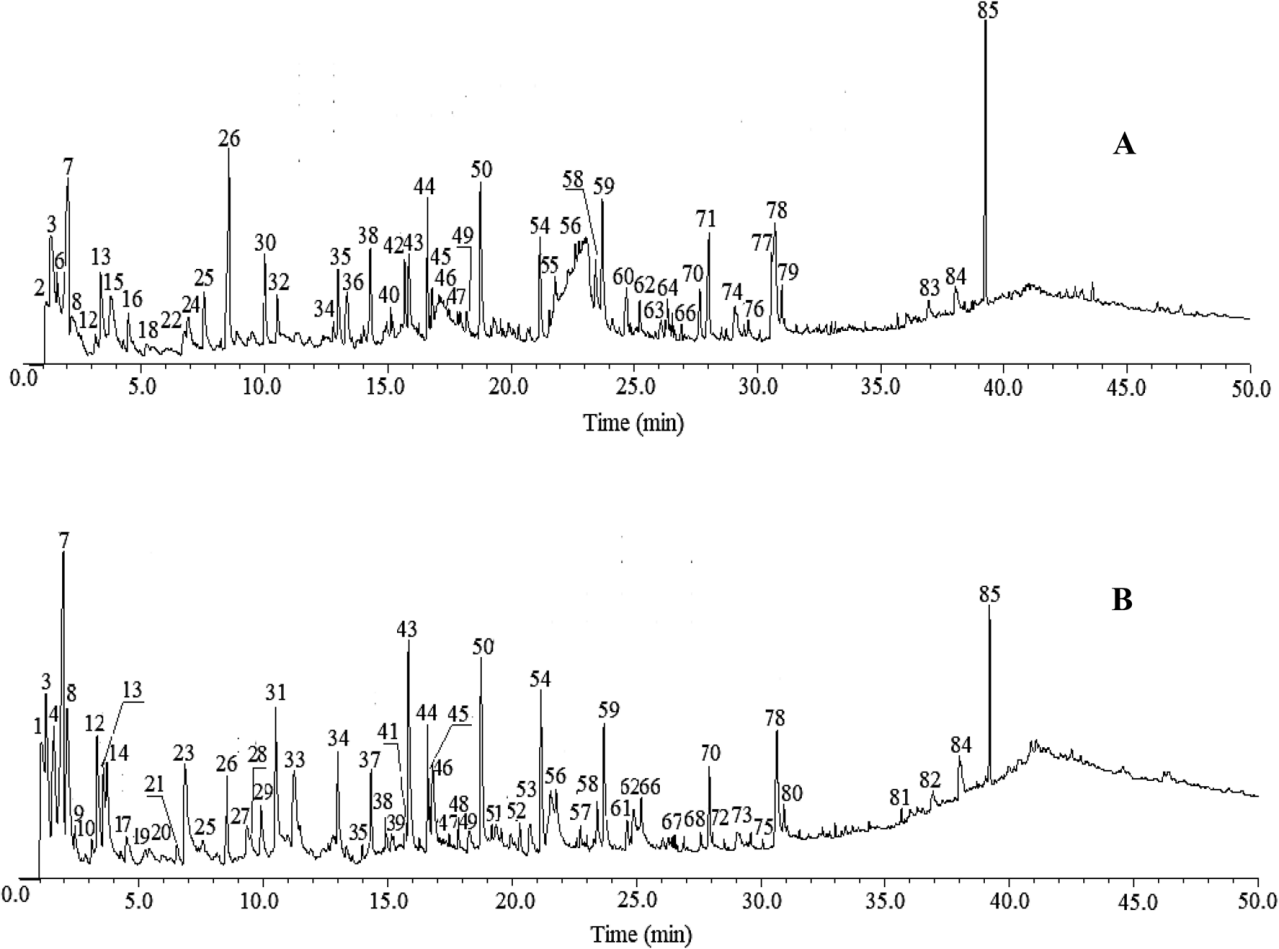
Py–GC/MS chromatogram of the sapwood (**A**) and heartwood (**B**) of the *Tectona grandis* wood.

The highest peak intensity in the sapwood pyrogram (Fig. 1A) correspondes to acetic acid (peak 7), phenol, 4-ethenyl-2-methoxy (peak 43), isoeugenol (peak 50), 3',5'-dimethoxyacetophenone (peak 54) and squalene (peak 85). In the heartwood region (Fig. 1B), the highest peak intensity corresponded to acetic acid (peak 7), cyclohexanecarboxaldehyde, 4-methoxy (peak 26) and squalene (peak 85). The most of these compounds, like acetic acid are derivatives of cellulose and hemicellulose, major components of wood, the degradation of lignins generate phenolic compounds, finally, extractives are not very representative and are divided into several compounds, thus, their derivatives are not very representative in the mass of compounds evaluated^16^.

The highest peak intensity corresponding to acetic acid (peak 7), phenol, 4-ethenyl-2-methoxy (peak 43), isoeugenol (peak 50), 3',5'-dimethoxyacetophenone (peak 54) and squalene (peak 85) from the lignin pyrolysis products, which are more abundant and easier to identify than carbohydrate derivatives^15^. The mass spectra with more abundant molecular ions are due to the greater thermal stability of the lignin aromatic compounds during pyrolysis and to the impact of electrons from the mass spectrometer^15^. It is easier to identify lignin-derived products because its pyrolysis releases a mixture of phenol derivatives preserving the aromatic ring, with the original methoxyl groups and a part or all of the propane side chain^20^. Furthermore, pyrolysis can cause multiple rearrangements in the carbohydrates resulting in isomers lacking molecular ions and, therefore, difficult to interpret^21^.

Levoglucosan was the main pyrolytic product formed from the heartwood sample (26.65%).

The formation of levoglucosan is due to the conversion of cellulose, hemicellulose, lignin and extractives from biomass into sugar, acids and furans, compounds with larger areas^7^. Sugars can be used as a carbon source, in fermentation, to produce compounds of industrial interest, such as citric acid, ethanol, 5-hydroxymethylfurfural, furans, aromatic hydrocarbons and lipids^22,23^.

The biphenyl compounds (1,1'-biphenyl-3,4,4'-trimethoxy-6'-formyl) and anthraquinone, 2-methyl, from the naphthoatraquinone group were identified at the end of the chromatogram of the sapwood and heartwood (Fig. 1).

The identification of biphenyl (1,1'-biphenyl-3,4,4'-trimethoxy-6'-formyl) and anthraquinone, 2-methyl compounds from the naphthoatraquinone group, at the end of the chromatogram for the sapwood and heartwood are due to the difficulty of fragmentation into small components and because they are derivatives of lignin. This compound, during pyrolysis, releases a mixture of phenolic derivatives preserving the aromatic ring, with the original methoxyl groups, and a part, or all, of the side chain of propane^20^. These components, with antifungal and bactericidal properties, protect the plant against xylophagous organisms, increasing the durability of its wood^24,25^. In addition, anthraquinones can replace synthetic dyes^26–28^.

### Syringyl/guaiacyl ratio

The syringyl/guaiacyl (S/G) ratio was 0.51 ± 0.010 and 0.50 ± 0.012 for the sapwood and heartwood respectively. The 4-vinylsyringol, trans-coniferyl alcohol and coniferaldehyde, 4-vinylguaiacol, 4-methylguaiacol, vanillin and sinapinaldehyde were the main compounds derived from lignin.

The S/G ratio of the *T. grandis* heartwood and sapwood was lower than that of the 70-year-old teak wood from the DBH position, 0.8 for heartwood and sapwood with total lignin content of 35.4% for the sapwood and 37.3% for the heartwood^15^ and for the 12 years old teak trees with a total lignin content of 31% and a S/G ratio of 0.72^29^ and of *Eucalyptus* spp. used for Kraft Pulp production, with an S/G from 2.0 to 3.2^30^. Differences between the S/G ratio values may be due to the growth stage (age), climatic factors and exposure to microorganisms, insects and pests^31^. The S/G ratio is important for the pulp industry, as the alkaline hydrolysis reactions of lignin fragmentation vary with the ratio between syringyl units^29^. The chemical structure of syringyl lignin is less condensed and more favorable to delignification by Kraft cooking liquor due to the absence of the C5 reactive carbon available for reaction in the polymerization step of lignin biosynthesis^32^. In addition, the high content of guaiacyl units indicates a more condensed lignin (greater number of bonds between C–C units), increasing the thermal resistance of the wood^31^. The characterization of lignin by pyrolysis (Py-GC/MS) is an effective, faster and cheaper method with lower consumption of reagents, analysis time and higher precision compared to traditional ones^12^.

## Conclusion

A total of 85 compounds, 51 in sapwood and 62 in heartwood, were identified in the pyrolyzed of teak wood samples. The area and retention time of carbohydrate-derived compounds were lower with lignin derivatives and extractives appearing at the middle and at the end of the pyrogram. The highest intensity peaks in the sapwood pyrogram corresponded to acetic acid, phenol, 4-ethenyl-2-methoxy, isoeugenol, 3',5'-dimethoxyacetophenone and squalene and those in the heartwood to acetic acid, cyclohexanecarboxaldehyde, 4-methoxy and squalene. The compounds found can be used in the manufacture of paints, dyes, synthetic silks, cellulose acetate, enhancing the use of teak wood. The syringyl (S)/guaiacyl (G) ratio of heartwood and sapwood were 0.51 and 0.50, respectively. Gas chromatography with mass spectrometry is a useful, cheaper and fast methodology, and can be applied with small quantities of material.

## Data Availability

The datasets generated and analysed during the current study are available from the corresponding author on reasonable request.
